# Evaluating visitor perception and spatial preferences of various museums based on machine learning from 2016 to 2024

**DOI:** 10.1371/journal.pone.0327112

**Published:** 2025-07-11

**Authors:** Yuandi Jiang, Kalyna Pashkevych, Shibo Bi

**Affiliations:** 1 Kyiv National University of Technologies and Design, Kyiv, Ukraine; 2 Shaanxi University of Science and Technology, Xi’an, People’s Republic of China; 3 School of Design art & Media, Nanjing University of Science and Technology, Nanjing, People’s Republic of China; Chulalongkorn University, THAILAND

## Abstract

Museum architecture is essential for preserving cultural heritage. Understanding the spatio-temporal evolution of visitor preferences, image perceptions, and driving factors is vital for promoting cultural development. However, traditional methods such as questionnaires and interviews face challenges in elucidating how exhibition layouts, environmental facilities, and service quality affect visitor experience and satisfaction. In this study, 30 museums in 6 categories were selected as samples, and over 64,000 public online reviews from Dianping and Ctrip were selected as data sets. Kernel density and standard deviational ellipse methods revealed the spatio-temporal evolution of museum space preferences (2016–2024). TF-IDF and LDA algorithms identified key image perception themes. Visitor satisfaction was then evaluated with SnowNLP sentiment analysis to examine the dynamic correlation between the perception themes and satisfaction. The findings showed: 1) Museum visitors were highly concentrated in eastern coastal regions, with spatial distribution evolving from single-core to multi-core clusters, gradually expanding into central areas (e.g., Henan, Hubei, Shaanxi). 2) Museum image perception has shifted from object-centered to more human-centered experiences, with significant differences across the various categories. 3) Over 75% of visitors reported positive experiences, with ethnography museums showing the highest satisfaction in 2024 (*Pro* = 0.922), whereas history museums consistently had the lowest. 4) Satisfaction drivers were dynamic, with 85.26% of perception themes significantly correlated with satisfaction (*p* < 0.01), with rich collections, distinctive features, immersive experiences, and diverse visitation forms identified as the primary contributors to positive visitor experiences. Based on the comprehensive perspective of typology and spatio-temporal dynamic evolution, this study not only provides empirical support for museum space optimization, but also provides new ideas and strategies for functional research and methodological insights of public spaces.

## 1 Introduction

Museums, as vital carriers of cultural heritage, serve as crucial bridges between history, culture, and the public [1]. With the acceleration of globalization and urbanization, museums have evolved beyond their traditional roles in cultural preservation and education, becoming essential spaces for public cultural experiences, interaction, and social memory [2]. The architectural layout, image shaping, and management strategies of museums not only determine the effectiveness of cultural information dissemination but also profoundly influence the experiences and perceptions of visitors [3]. In recent years, the number of museums worldwide has increased significantly. In China alone, more than 200 new museums were added in 2023, with an annual visitor count exceeding 1.2 billion [4]. However, despite this increase, existing research on the spatial preferences and image perceptions of museums in different spatiotemporal contexts remains insufficient [5,6].

Generally, museum space research is divided into two dimensions: “building-object” and “visitor-subject” perspective. First, studies have focused on museum architectural design [7,8], spatial layout [9] and operational management [10], employing GIS to analyse the spatial distribution and kernel density characteristics of museums [11]. For example, Shen et al. (2024) suggested that integrating digital technologies into museum space design can enhance visitors’ perceptions, thus facilitating the effective transmission of cultural information [3]. Although these studies offer valuable insights into museum design and spatial optimization, most of them focus on a single point in time and lack dynamic analysis of the spatio-temporal evolution of museum spaces [8,9]. As long-term cultural spaces, museums’ functions and usage patterns evolve with urbanization and sociocultural changes. However, current research is inadequate [10].

Second, in terms of the visitor experience, affective cognition analysis is often used to measure visitors’ emotional tendencies (e.g., positive or negative) toward museums to assess their satisfaction levels [12,13]. Among them, investigating visitors’ psychological behavior [14], motivations and needs [15] and image perception [16] to evaluate museum satisfaction [17] and its influencing factors [18] has become a research hotspot. For example, Wen et al. (2024) and Ceccarelli et al. (2024) reported that the functional layout and cultural display forms of museums directly affect the public spatial experience and local cultural identity, respectively [2,18]. In these studies, traditional methods, such as questionnaires and interviews, remain key approaches for exploring how the exhibition layout, environmental facilities, and service quality influence the visitor experience and satisfaction [19,20]. For example, Chen et al. (2020), based on a survey of 740 respondents, found that long queue times and insufficient lighting significantly affected the visitors’ experience at the Shaanxi History Museum [19]. Although these studies reveal some behavioral preferences of visitors, studies based on limited samples have failed to capture the diverse perceptions of visitors across different types of museums [21,22]. More importantly, the dynamic changes in space image perception and satisfaction of museums across different spatio-temporal contexts have not been revealed [12]. This limits understanding of the differences among various types of museums and hinders the development of more targeted museum space design strategies.

In recent years, with the widespread use of digital technology and social media platforms, studies have increasingly recognized the potential of using large-scale social media texts to assess visitor experiences and perceptions [23–26]. Descriptive nouns and action verbs in texts are often used to perceive the image of a destination, whereas emotionally charged words reflect visitors’ positive or negative emotional tendencies, which are used to measure satisfaction levels [25]. For example, Arnaboldi et al. (2021) used the SOR model to analyse visitor online reviews, revealing differences in experiences among various archaeological museums [27]; Based on the Weibo data of Beijing, Xia et al., (2020) reveals the socio-cultural and functional characteristics of the city by understanding the perceived preferences of different visitors to the city’s image [28]. However, these studies focused primarily on macrolevel analyses of city image and tourism experience [27,29] and lacked an in-depth exploration of museum spatial preferences and image perceptions, particularly national-scale and museum-type differences [13,30].

Overall, several research gaps exist: 1) Museum space studies are often limited to static perspectives and fail to consider the complexity of spatial preferences and functional changes during spatiotemporal evolution [12,31,32]. 2) Traditional methods (e.g., questionnaires, structured interviews, etc.) are constrained by sample size and data collection methods and do not explore the diverse perceptions and experiences of visitors across different periods and regions from a typological perspective [28,33]. In contrast, social media-based big data and machine learning methods can overcome these limitations by extracting more comprehensive visitor feedback and perception changes from vast datasets [34,35]. 3) Image perception and satisfaction levels are often studied separately [13], and their complex relationships have not yet been systematically explored.

To address these gaps, this study combines spatiotemporal evolution analysis with machine learning techniques and collects online review data from 30 different types of museums in China. Using kernel density analysis, standard deviation ellipse, the term frequency and inverse document frequency (TF-IDF) algorithm, the latent dirichlet allocation (LDA) topic model, and SnowNLP sentiment analysis, this study systematically reveals the spatiotemporal dynamics of spatial preferences, image perceptions, and satisfaction levels across different museums. This study not only offers scientific design optimization recommendations for museum managers to promote the preservation and dissemination of cultural heritage but also provides methodological insights for research on other public cultural spaces. This study aims to answer the following key questions: **1)** What are the spatio-temporal evolution characteristics of spatial preferences among visitors to different types of museums from 2016–2024? **2)** What are the core themes of spatial image perception in different types of museums, and what are their dynamic trends? **3)** What is the relationship between space satisfaction level and space image perception themes? **4)** What strategies can be employed to optimize museum space design to enhance space experience?

## 2 Methodology

### 2.1 Data sources and preprocessing

This section includes three steps: obtaining, cleaning, and segmenting online text data to prepare for subsequent analysis.

**Fig 1:**
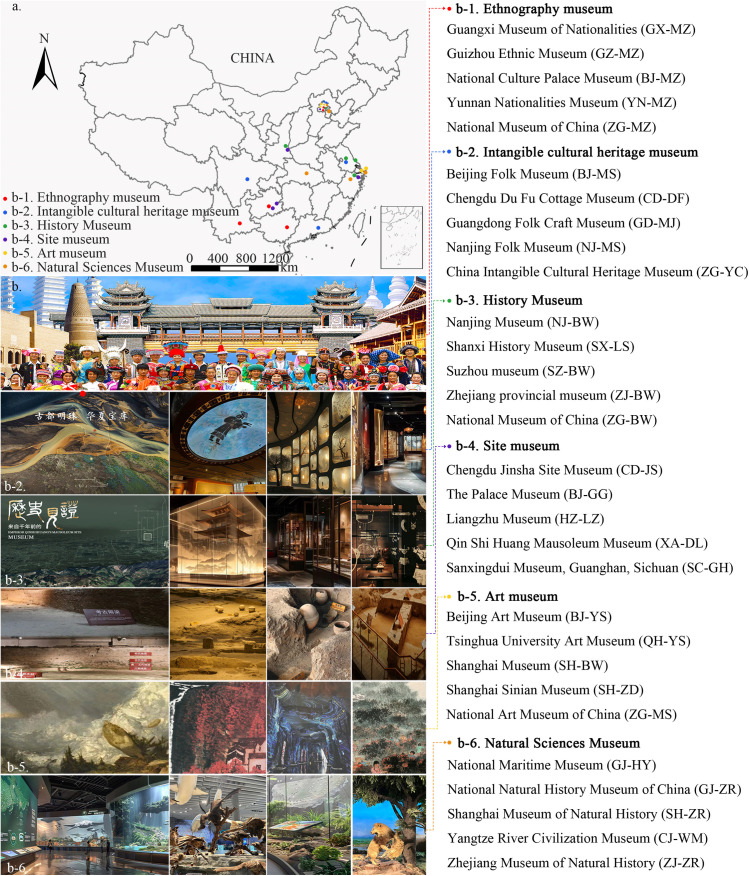
Study subjects and spatial distribution: spatial locations (a), diagram of different museum types (b).

First, online text data were obtained. This study selected the top 5 museums from each category of the latest Chinese Museum Trending Searches (CMTS) ranking, published in April 2024, which categorized museums into 6 types: intangible cultural heritage, history, ethnography, site, art, and natural science (Total 30) (Fig 1). The data sources include open-source public online reviews from Dianping (http://www.dianping.com) and Ctrip (https://www.ctrip.com). Python were used to scrape and record relevant review data from three periods (2016, 2020, and 2024), capturing details such as user ID, URL, review content, and review date. Initially, the dataset comprised over 64,000 online reviews containing more than 5,900,000 Chinese characters. All data collection and analysis procedures strictly complied with the terms and conditions of the respective platforms.

Second, data cleaning was performed to ensure data quality. Specifically, invalid reviews (e.g., advertisements, venue descriptions) and meaningless or duplicate reviews were removed, resulting in 56,320 valid reviews with a total of over 5,242,700 Chinese characters. Subsequently, Python’s Jieba library was used for word segmentation, and stop words (e.g., “my,” “haha,” and other non-informative terms) were removed, resulting in approximately 76,370 valid phrases.

### 2.2 Methods

**Fig 2:**
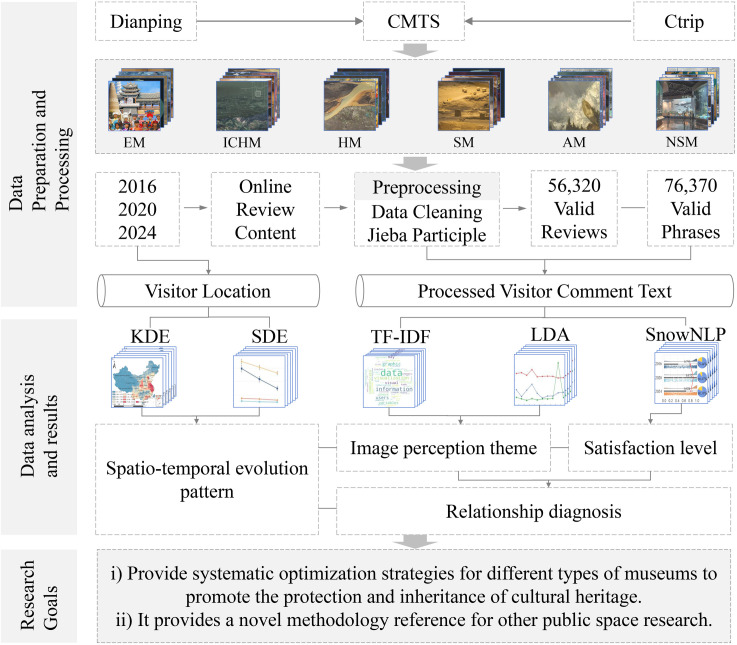
Research framework. EM, ICHM, HM, SM, AM, and NSM are 6 types of museums (Ethnography, intangible cultural heritage, History, Site, Art, Natural Sciences museums); CMTS: Chinese Museum Trending Searches; KDE: kernel density estimation; SDE: Standard deviational ellipse.

In this study, GIS spatial analysis and machine learning models (e.g., LDA topic modeling, etc.) were integrated to explore the spatio-temporal evolution of visitor preferences, image perception themes and satisfaction levels in 30 museums representing six types (Fig 2). This comprehensive approach captures the dynamic evolution of visitor behaviors and spatial patterns from a spatio-temporal perspective, overcomes the limitations of static traditional approaches, and provides powerful quantitative insights from a wide array of online reviews.

The specific steps of the study are as follows: First, the spatio-temporal evolution patterns are revealed. Using the review locations, spatial positioning was performed with a GIS platform, and kernel density analysis along with the standard deviation ellipse method was employed to explore the spatial distribution patterns and evolution of visitor preferences in six types of museums over different periods (2016, 2020 and 2024). Second, the image perception themes were assessed. By performing TF-IDF analysis on online review content, the main features of visitor preferences for 30 museums were identified. The LDA model was then used to determine the image perception themes and their evolution for different museum types from 2016–2024. Third, the satisfaction levels are evaluated. On the basis of the online review content, SnowNLP sentiment analysis was conducted to analyse the sentiment scores of visitors to six types of museums, which were used to measure their satisfaction with museum spaces [36]. Finally, the influencing factors are revealed. Pearson correlation analysis was used to explore the impact of image perception themes on museum satisfaction, identifying the factors that contribute to positive or negative visitor experiences. Consequently, this study, based on an online review database and from spatio-temporal and typological perspectives, construct a comprehensive evaluation framework of visitor preferences, image perception themes, satisfaction levels, and influencing factors in museums. The resulting insights provide evidence for museum space optimization.

#### 2.2.1 Kernel density estimation.

Kernel density estimation (KDE) is a nonparametric method used to estimate the density of point data within a given area, revealing the spatial clustering of these points [37]. In this study, the geographic locations of museum visitors were marked as point data on the GIS platform. The KDE method was applied to quantify the spatial distribution characteristics of visitors to different types of museums in 2016, 2020, and 2024 (Equation (1)).


$$\beta \left(x\right)=\frac{1}{nh}\sum {\mathrm{\backslash nolimits}}_{i=1}^{n}k\left(\frac{x-x_{i}}{h}\right)$$ (1)

where *β* (x) represents the kernel density value of the point data, *n* represents the number of visitors from each province, $k\left(\frac{x-x_{i}}{h}\right)$ is the kernel function, *h* is the bandwidth (*h* > 0), and (*x-x*_*i*_) represents the distance between sample point *x* and sample point *x*_*i*_.

#### 2.2.2 Standard deviational ellipse.

The standard deviational ellipse (SDE) is used to reveal the directionality, centroid, and deviation in the distribution of point data [38]. In this study, the SDE was applied to calculate the spatial evolution characteristics of visitors to different types of museums from 2016 to 2024. The main parameters include the centroid (Equation (2)), orientation (Equation (3)), ellipse area (Equation (4)), and major (*x*) and minor (*y*) axes (Equations (5) and (6)). The centroid indicates the central location of visitor aggregation, while the area of the SDE reflects the distribution range of visitors, and the orientation shows the direction of distribution spread. The ratio of the major to the minor axis is known as the eccentricity; the larger (or smaller) the eccentricity is, the more (or less) concentrated the distribution of visitors [39].


$$\overset{―}{X},\overset{―}{Y}=\left[\frac{\sum_{i=1}^{n}w_{i}x_{i}}{\sum_{i=1}^{n}w_{i}},\frac{\sum_{i=1}^{n}w_{i}y_{i}}{\sum_{i=1}^{n}w_{i}}\right]$$ (2)

$$\tan\theta =\frac{\left(\sum_{i=1}^{n}w_{i}^{2}{\hat{x}}_{i}^{2}-\sum_{i=1}^{n}w_{i}^{2}{\hat{y}}_{i}^{2}\right)+\sqrt{{\left(\sum_{i=1}^{n}w_{i}^{2}{\hat{x}}_{i}^{2}-\sum_{i=1}^{n}w_{i}^{2}{\hat{y}}_{i}^{2}\right)}^{2}+4\sum_{i=1}^{n}w_{i}^{2}{\hat{x}}_{i}^{2}{\hat{y}}_{i}^{2}}}{2\sum_{i=1}^{n}w_{i}^{2}{\hat{x}}_{i}^{2}{\hat{y}}_{i}^{2}}$$ (3)

S=π∂x∂y (4)

$$\partial x=\frac{\sqrt{2\sum_{i=1}^{n}\left(w_{i}{\hat{x}}_{i}\cos\theta -w_{i}{\hat{y}}_{i}\sin\theta \right)}}{\sum_{i=1}^{n}w_{i}^{2}}$$ (5)

$$\partial y=\frac{\sqrt{2\sum_{i=1}^{n}{\left(w_{i}{\hat{x}}_{i}\sin\theta -w_{i}{\hat{y}}_{i}\cos\theta \right)}^{2}}}{\sum_{i=1}^{n}w_{i}^{2}}$$ (6)

where ${\hat{x}}_{i}=x_{i}-\overset{―}{x}$ and ${\hat{y}}_{i}=y_{i}-\overset{―}{y}$ represent the coordinate deviations of each province from the mean center; (*x*_*i*_*, y*_*i*_) represent the latitude and longitude coordinates of each provincial center; *w*_*i*_ represents the exact latitude and longitude of the location of visitor; $\bar{x},\bar{y}$ represents the mean center coordinate; tanθ represents the ellipse orientation; $\partial_{x}$ and $\partial_{y}$ represent the standard deviations of the ellipse’s *x-* and *y*-axes; and *S* represents the area of the standard deviational ellipse (km^2^).

#### 2.2.3 *TF‒IDF* analysis.

Words are the basic units of a text, and the frequency with which words appear in online reviews can reveal visitors’ focus on the image characteristics and various perception elements of destination [40]. The *TF-IDF* algorithm is a commonly used text vectorization method composed of term frequency (*TF*) and inverse document frequency (*IDF*) (Equation (7)) and is used to evaluate the importance of words in online texts [41]. TF represents the frequency of a word in a document (Equation (8)), and IDF represents the word’s ability to distinguish categories (Equation (9)). In this study, the *TF-IDF* algorithm was used to extract keywords and their weights from online reviews, revealing spatial image perceptions of different types of museums.


TF−IDF=TF×IDF (7)

$$TF(t)=\frac{m\left(t\right)}{M}$$ (8)

$$IDF=\log\left(\frac{N}{N\left(t\right)+1}\right)$$ (9)

where *t* represents a specific phrase in the document; *m(t)* indicates the total number of occurrences of phrase *t* in the document; *M* represents the total number of phrases in the document, which is 76,370 in this study; *N* is the total number of valid comments, which is 56,320 in this study; and *N(t)+1* is the number of comments containing the phrase *t*.

#### 2.2.4 Latent dirichlet allocation topic mining.

First, the optimal number of topics (*K*) was determined. This was verified by calculating the coherence and perplexity of the online text data from six types of museums between 2016 and 2024 using the Matplotlib tool in Python [42]. Perplexity refers to the certainty of the mapping between image topics and phrases, whereas coherence measures the semantic similarity of high-frequency words corresponding to each topic. In this study, higher coherence values and lower perplexity values were used to determine the number of image perception topics for each type of museum across different periods.

Second, on the basis of the determined *K* value, the LDA model, as an unsupervised machine learning technique, was used to construct a three-layer Bayesian “document-topic-word” structure to identify latent image perception topics in a large document collection or corpus [41]. This model was used to analyse the latent key information on visitors’ spatial experiences of six types of museums in online reviews over different periods and further classify museum space image perception topics [42] (Equation 10). Finally, on the basis of the identification of the logical relationships among the top 20 phrases in each topic, these topics were systematically summarized and categorized into different image perception themes.


R(W/WD\nulldelimiterspaceD)=∑\nolimitskR(W/WT\nulldelimiterspaceT)×R (10)

where *D* represents the review documents; *T* represents the topics; *K* represents the number of topics; *R(W/D)* represents the frequency of phrase occurrence in each review; *R(W/T)* represents the probability of each characteristic word appearing in a given topic; and *R* represents the distribution probability of different image perception topics in the online review documents for various types of museums across different periods and is used to measure the strength of each topic [41].

#### 2.2.5 Sentiment analysis with SnowNLP.

Sentiment analysis refers to the process of analysing, processing, inducing and reasoning on subjective texts with emotional color through the use of natural language processing technology, and its goal is to analyse public emotional tendencies and opinions on the research object [43]. In this study, the SnowNLP analysis method, which is based on an emotional dictionary, has strong stability and excellent vertical effects [44]. The basic principle is as follows: suppose that the classification of sentiment analysis includes positive evaluation (*c*_*1*_) and negative evaluation (*c*_*2*_) and that each review has n mutually independent text spaces *W*_*1*_*..., W*_*n*_. The naive Bayes formula is used to calculate the conditional probabilities of the positive evaluation *Pro (C*_*1*_ *| W*_*1*_*..., W*_*n*_) and the negative evaluation *Pro (C*_*2*_ *| W1..., Wn)*. These values were calculated via Formula (11).


$$Pro\left(Ci|W_{1},...,W_{n}\right)=\frac{P\text{ro}\left(W_{1},\ldots ,W_{n}|C_{i}\right)P\left(C_{i}\right)}{Pro\left(W_{1},\ldots ,W_{n}\right)}$$ (11)

On the basis of the total probability Formula *Pro(B)=Pro(B|A)Pro(A)+Pro(B|A’)Pro(A’)*, *Pro (W1..., Wn)* is expressed as Equation (12), which can then be converted to Equation (13):


$$Pro\left(W_{1},\ldots ,W_{n}\right)=Pro\left(W_{1},\ldots ,W_{n}|C_{1}\right)Pro\left(C_{1}\right)+Pro\left(W_{1},\ldots ,W_{n}|C_{2}\right)Pro\left(C_{2}\right)$$ (12)

$$Pro\left(C_{i}|W_{1},\ldots ,W_{n}\right)=\frac{Pro\left(W_{1},\ldots ,W_{n}|C_{i}\right)}{Pro\left(W_{1},\ldots ,W_{n}|C_{1}\right)Pro\left(C_{1}\right)+\mathrm{Pr}o\left(W_{1},\ldots ,W_{n}|C_{2}\right)Pro\left(C_{2}\right)}$$ (13)

The calculated probability value (*Pro*) ranges between 0 and 1. When *Pro* is closer to 1, the positive direction is represented, and when *Pro* is closer to 0, the negative direction is represented. According to the probability score, emotional tendency was divided into three different categories: positive (*Pro* > 0.6), neutral (0.6 ≥ *Pro* ≥ 0.4), and negative (*Pro* < 0.4) [29]. In this study, the *Pro*-value was used to measure visitor satisfaction levels with different types of museums across different periods.

#### 2.2.6 Pearson correlation analysis.

Pearson correlation analysis was used to further explore the potential relationships between space image perception themes and satisfaction levels across different periods, providing strategic insights for enhancing the spatial experience of museums (Equation (14)). The *p* value was used to test the significance level (Equation (15)). In this analysis, the probability of occurrence of characteristic words within each image perception topic *(R(W/T))* was used as the explanatory variable, and the visitor satisfaction level (*Pro* value) was used as the dependent variable.


$$r=\frac{\sum_{i=1}^{n}\left(X_{i}-\overset{―}{X}\mathrm{\backslash rightleft}(Y_{i}-\overset{―}{Y}\right)}{\sqrt{\sum_{i=1}^{n}{\left(X_{i}-\overset{―}{X}\right)}^{2}}\sqrt{\sum_{i=1}^{n}{\left(Y_{i}-\overset{―}{Y}\right)}^{2}}}$$ (14)

$$p=\frac{r\sqrt{n-2}}{\sqrt{1-r^{2}}}$$ (15)

where *r* represents the correlation coefficient; *n* represents the sample size; *X*_*i*_ and *Y*_*i*_ represent the topic probability value and visitor sentiment score of the ith study unit, respectively; and $\bar{x}$ and $\bar{y}$ represent the mean values of the topic probability and visitor sentiment score, respectively. The *p* value represents the significance level, which is typically set at 0.05. If *p* < 0.05, there is a significant relationship between the topic and visitor satisfaction [45].

## 3 Results

### 3.1 Spatiotemporal distribution pattern of museum visitor preferences

#### 3.1.1 Overall spatial distribution characteristics.

**Fig 3:**
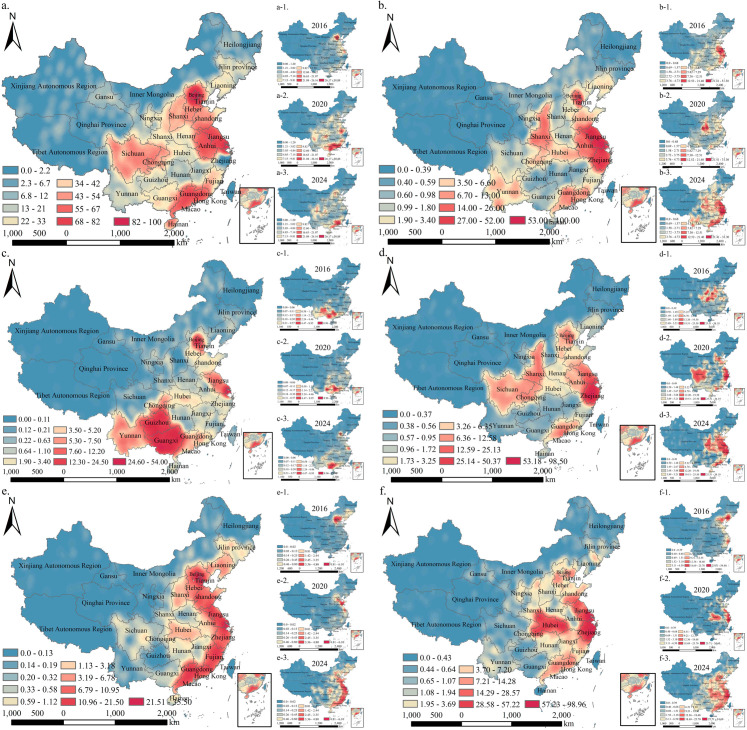
Kernel density analysis of visitor preferences for different museum types. a–f represent intangible cultural heritage, history, ethnography, site, art, and natural science museums, respectively. Taking (a) as an example, a-1, a-2, and a-3 represent kernel density maps for visitors in 2016, 2020, and 2024, respectively.

The spatial distribution of visitor preferences for the six types of museums is shown in Fig 3. Excluding northeastern and western China, other regions display varying degrees of preference for the six museum types, overall showing a “multicore” spatial distribution.

Overall, visitors from Beijing and the eastern coastal regions of Jiangsu and Zhejiang show consistently high preference for all six museum types, especially for intangible cultural heritage museums (*β *= 82.00–100.00) and history museums (*β *= 53.00–100.00). In contrast, the spatial clustering patterns of visitors for other types of museums shows significant regional differences. Specifically, ethnography museums exhibit high-density visitor clusters in ethnic minority regions such as Guizhou and Guangxi (*β* = 24.60–54.00), whereas visitors to art museums are primarily from developed coastal regions such as Jiangsu and Guangdong (*β* = 6.79–35.50), with secondary clusters forming in central and western regions such as Hubei and Shanxi (*β* = 1.13–6.78). Additionally, visitors to site museums (*β* = 3.26–25.13) and history museums (*β* = 3.50–26.00), which evoke strong historical experiences, are concentrated primarily in central and western regions such as Hubei, Sichuan, and Shaanxi.

#### 3.1.2 Spatiotemporal evolution of museum visitor distribution patterns.

**Fig 4:**
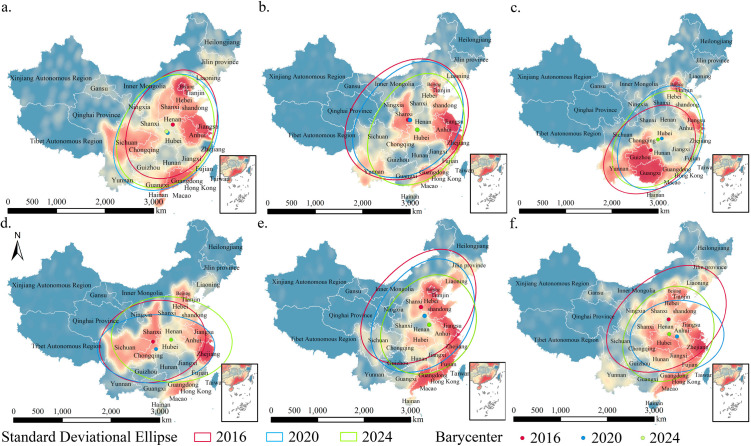
Standard deviational ellipse distribution and centroid shift trajectories of visitor regions for different museum types. a–f represent intangible cultural heritage, history, ethnography, site, art, and natural science museums, respectively.

**Fig 5:**
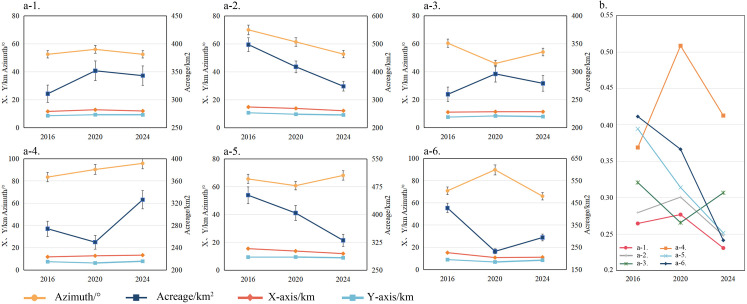
Long and short axes, azimuth, and area data of standard deviational ellipses (a) and eccentricity changes (b). a-1 to a-6 represent Intangible Cultural Heritage, History, Ethnography, Site, Art, and Natural Science museums, respectively.

Overall, from 2016 to 2024, the spatial distribution pattern of visitor preferences for various museum types evolved from a predominantly “single-core” pattern to a more dispersed “multi-core” configuration, with a notable decline regional connectivity observed in 2020 (Figs 3–5).

Specifically, the centers of museum visitor distribution remained concentrated in the central and western regions (e.g., Henan, Hubei, Shaanxi), with varying degrees of shifts over time. For intangible cultural heritage and natural science museums, the clockwise shift in the center of the visitor distribution gradually diminished, whereas the center for art museums consistently moved southeastward. Additionally, the overall diffusion direction of the visitor distribution shifted from “northeast‒southwest” to “east‒west” on the basis of the ellipsoid area and azimuth. Except for history and site museums, which experienced a reduction in visitor coverage (greater than 15%), the remaining four museum types showed an initial expansion followed by contraction in the standard deviation ellipsoid area. Finally, except for site museums, the degree of outwards diffusion for other museum types showed fluctuating growth, particularly for art museums, where the degree of diffusion increased steadily, with the flattening ratio decreasing from 0.39 in 2016 to 0.26 in 2024.

### 3.2 High-frequency phrase analysis of museum spatial perception

High-frequency phrases are concentrated around a few core words, indicating a high degree of similarity in spatial perceptions of museum spatial preferences. Specifically, 1) 56,320 review texts contained 76,370 valid words. Words that appeared only once accounted for 46.11% of the total. There were 35 high-frequency words with more than 4,000 occurrences, mainly nouns (19) and verbs (11) (Table 1). Nouns are related primarily to museum attributes and functions, such as “history” (11,971), “culture” (11,070), and “exhibits” (10,735), indicating that visitors place importance on the core elements of museums. Frequent mentions of words such as “China” (8,468) and “Ethnic” (3,279) suggest that visitors view museums as significant windows for the dissemination of national and ethnic images. 2) Verbs mainly reflect visitor behaviors and attitudes, such as “reservation” (15,153), “visit” (11,214), and “like” (3,591). 3) Adjectives mostly describe the atmosphere of the museum and personal feelings, such as “advanced” (8,668) for reservations and “special” (3,748) for the museum’s atmosphere and displays.

**Table 1:** Distribution of high-frequency words in online reviews in the top 35 words.

Words	Frequency	*TF‒IDF*	Part of speech	Words	Frequency	*TF‒IDF*	Part of speech
Museum	34761	1.91	Noun	Special	3748	0.37	Adjective
Reservation	15153	0.83	verb	Exhibition hall	3745	0.36	Noun
Guided tour	13381	0.73	verb	Like	3591	0.34	verb
History	11971	0.66	Noun	Hour	3542	0.32	Noun
Exhibition	11914	0.65	Noun	Art	3430	0.30	Noun
Visit	11214	0.61	verb	Ethnic	3279	0.30	Noun
Culture	11070	0.61	Noun	Children	3224	0.29	Noun
Exhibit	10735	0.59	Noun	Transportation	3191	0.27	Noun
Place	9105	0.50	Noun	Suggestion	3184	0.25	verb
Early/Advance	8668	0.48	Adjective	Content	3177	0.24	Noun
Time	8600	0.47	Noun	Check-in	3124	0.24	verb
China	8468	0.46	Noun	Take photos	3110	0.22	verb
Architecture	8364	0.46	Noun	Experience	3082	0.21	verb
Worthy	8183	0.45	Adjective	Recommend	3057	0.21	verb
Artifact	8019	0.44	Noun	Site	3054	0.21	Noun
Ticket	7756	0.43	Noun	Open	3047	0.20	Adjective
Experience	7633	0.42	verb	Design	3015	0.19	verb
Free	7247	0.40	Adjective				

In conclusion, spatial preferences for museums are primarily concentrated around key elements such as “reservation” and “historical culture,” reflecting visitors’ strong demand for ease of access and cultural richness in museums. Among these, the “reservation” aspect of museums has become a point of attention, likely because of the challenges posed by managing large numbers of visitors. Moreover, public emphasis on cultural imagery indicates that the historical and cultural value of museums plays a key role in enhancing their experience perceptions.

### 3.3 Identifying museum spatial perception and theme evolution

**Fig 6:**
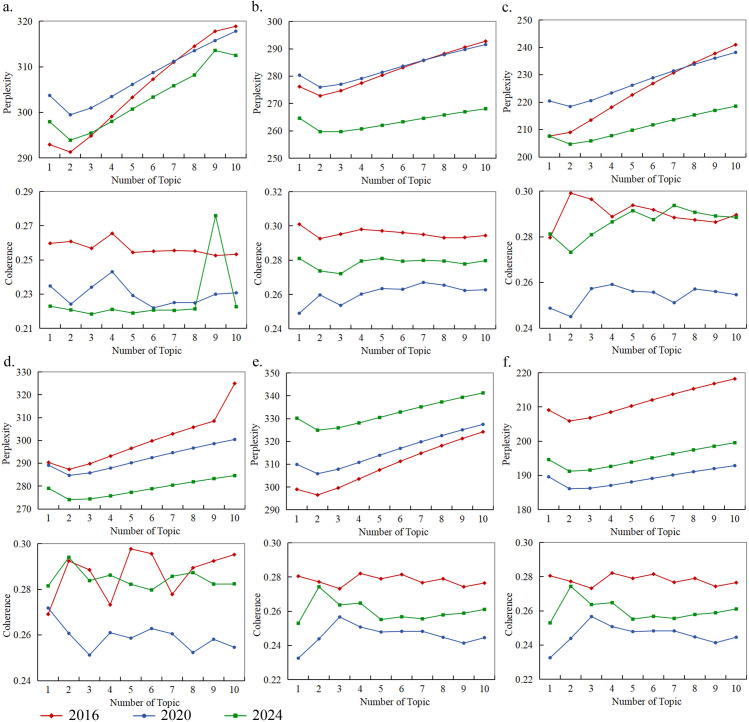
LDA topic perplexity and coherence testing. a–f represent intangible cultural heritage, history, ethnography, site, art, and natural science museums, respectively.

On the basis of topic coherence and perplexity tests (Fig 6), a total of 95 perception themes were identified for the six types of museums across three time periods (Table 2). Overall, public perceptions of museum spaces exhibited increasing diversity from 2016 to 2024 (with increasing *K* values). In 2024, intangible cultural heritage museums demonstrated the broadest range of perception themes (*K* = 9), whereas art museums consistently presented fewer perception themes across all periods (*K *= 4). Additionally, from 2016 to 2024, public perceptions differed significantly across museum types, with noticeable differences in the intensity and distribution of similar themes across different museum categories.

**Table 2:** Spatial image perception themes for the 6 museum types between 2016 and 2024; X_1_–X_95_ represent the codes of 95 spatial image themes.

Year	*K*	*Intangible cultural heritage museum*: Image perception theme (R)
2016	4	X_1_: Diversity of exhibit content (0.080), X_2_: Detailed exhibit descriptions (0.167), X_3_: Ticket cost-effectiveness (0.280), X_4_: Environment and cultural atmosphere (0.474)
2020	4	X_5_: Comfortable visiting environment (0.181), X_6_: Opening hours and reservation manageme (0.356), X_7_: Multi-dimensional interpretation services (0.110), X_8_: Historical environment and cultural atmospher (0.353)
2024	9	X_9_: Representation of architecture and folk culture (0.133), X_10_: Ticket cost-effectiveness (0.396), X_11_: Disorderly organization (0.015), X_12_: osting of special themed events (0.020), X_13_: Special cultural exhibitions and experiences (0.163), X_14_: Visitor interaction and digital experience (0.101), X_15_: Special events and festival experiences (0.085), X_16_: Multi-dimensional interpretation and knowledge transmission (0.019), X_17_: Spiritual sustenance (0.068)
Year	*K*	*History museum*: Image perception theme (R)
2016	4	X_18_: Quality and diversity of collections (0.122), X_19_: Reservation and queue management (0.573), X_20_: Visual impact of historical artifacts (0.105), X_21_: Impressive scale of the venue (0.201)
2020	7	X_22_: Environmental atmosphere (0.129), X_23_: Reservation and queue management (0.040), X_24_: Relevance of exhibits to historical value (0.068), X_25_: Cultural dissemination and heritage preservation (0.022), X_26_: Educational expansion for school-age children (0.013), X_27_: Variety of artifacts (0.234), X_28_: Interpretation services and cultural education (0.493)
2024	5	X_29_: Check-ins and social media sharing (0.546), X_30_: Richness of collections (0.063), X_31_: Noisy visiting environment (0.086), X_32_: Reservation and visitor flow management (0.117), X_33_: Cultural experiences and creative product development (0.188)
Year	*K*	*Ethnography museum*: Image perception theme (R)
2016	5	E_34_: Visitor flow and time management (0.177), X_35_: Display of ethnic cultural characteristics (0.098), X_36_: Venue size (0.163), X_37_: Reservation and geographical convenience (0.073), X_38_: Ethnic festivals and activity experiences (0.488)
2020	4	X_39_: Ethnic culture exhibition and interactive experience (0.104), X_40_: Educational functions and interpretation services (0.064), X_41_: Visitor flow and time management (0.444), X_42_: Ethnic culture exhibition and integration (0.387)
2024	7	X_43_: Brief introduction (0.490), X_44_: Short visiting time (0.027), X_45_: Tour route planning (0.045), X_46_: Check-ins and social media sharing (0.028), X_47_: Exhibition of ethnic clothing and traditional crafts (0.090), X_48_: Interaction between outdoor exhibits and natural landscapes (0.291), X_49_: Convenience of transportation and location (0.028)
Year	*K*	*Site museum*: Image perception theme (R)
2016	5	X_50_:Authenticity of archaeological site (0.227), X_51_:Spacious venue (0.037), X_52_:Visual impact of venue size (0.167), X_53_:Consistency between artifacts and their information (0.525), X_54_:Ticket cost-effectiveness matching exhibition quality (0.045)
2020	6	X_55_:Visitor flow and time management (0.164), X_56_:Grand and mysterious visual impact (0.191), X_57_:Heritage preservation and restoration display (0.246), X_58_:Relevance of exhibits to the theme (0.042), X_59_:Expression of city/national image (0.024), X_60_:Choice of interpretation services (0.332)
2024	8	X_61_:Disorderly visiting environment (0.035), X_62_:Size of the collection (0.062) X_63_:Stunning visual experience (0.082), X_64_:Interpretation services and interactive experience (0.358) X_65_:Authenticity of site displays (0.157), X_66_:Reservation and visitor flow management (0.131) X_67_:Check-ins and social media sharing (0.157), X_68_:Association with literary works (0.018)
Year	*K*	*Art museum*: Image perception theme (R)
2016	4	X_69_:Frequent changes of themed exhibitions (0.150), X_70_:Space suitable for parent-child activities (0.107), X_71_:Reservation and visit time management (0.661), X_72_:Rarity of masterworks (0.082)
2020	3	X_73_: Convenience of reservation and entry (0.485), X_74_: Multi-dimensional online visiting services (0.364), X_75_: Rich master-themed exhibitions (0.151)
2024	4	X_76_:Cultural derivative products (0.102), X_77_:Complicated reservation process (0.581), X_78_:Immersive scene construction (0.209), X_79_:Transmission of cultural knowledge (0.101)
Year	*K*	*Natural Sciences Museum*: Image perception theme (R)
2016	6	X_80_:Easy-to-understand interpretation of works (0.107), X_81_:Transmission of cultural knowledge (0.145), X_82_:Reservation and queue management (0.232), X_83_:Parent-child interaction experience (0.104), X_84_:Real specimen displays (0.323), X_85_:Comfortable venue environme (0.058)
2020	4	X_86_:Attraction and educational value of artifacts (0.208), X_87_:Stunning effect of natural and biological displays (0.067), X_88_:Visitor management and flow control (0.183), X_89_:Integration of modern technology (0.542)
2024	6	X_90_:Leisure social venue (0.024), X_91_:Supporting facilities and services (0.021), X_92_:Reservation and queue management (0.686), X_93_:Check-ins and social media sharing (0.037), X_94_:Effective parent-child educational experience (0.048), X_95_:Digital interactive experience and knowledge transmission (0.185)

Specifically, 1) the focus shifted from “objects” to “people.” From 2016 to 2020, spatial perception themes transitioned from the characteristics and visual impact of exhibits, such as “authentic specimens” (*R* = 0.323) and “visual impact” (*R* = 0.105), to themes related to the historical and cultural significance of the exhibits, such as “cultural dissemination and social sharing” (*R* = 0.353) and “city/nation image expression” (*R* = 0.024). In 2024, public placed more emphasis on personal experiences during their visits, particularly in aspects such as special cultural event participation, cultural product derivation (*R* = 0.188), interaction with outdoor exhibits and natural landscapes (*R* = 0.291), and immersive scene construction (*R* = 0.209). 2) Differences in spatial perception themes across museum types. Compared with academic research and collection functions, visitors to intangible cultural heritage, history, and site museums place greater emphasis on cultural education functions (knowledge dissemination) and the environment (visual impact). In contrast, visitors to Art, Ethnography, and Natural Science museums focused more on cultural festivals, local cultural identity, and recreational functions. 3) The intensity of similar perception themes varied across different museum types. For instance, the theme related to “reservation and ticket price value” was perceived more strongly in Art (*R* = 0.661) and History museums (*R* = 0.573) but was less prominent in Site museums (*R* = 0.164).

### 3.4 Analysis of museum spatial experience satisfaction

#### 3.4.1 Overall satisfaction levels of different museum types.

**Fig 7:**
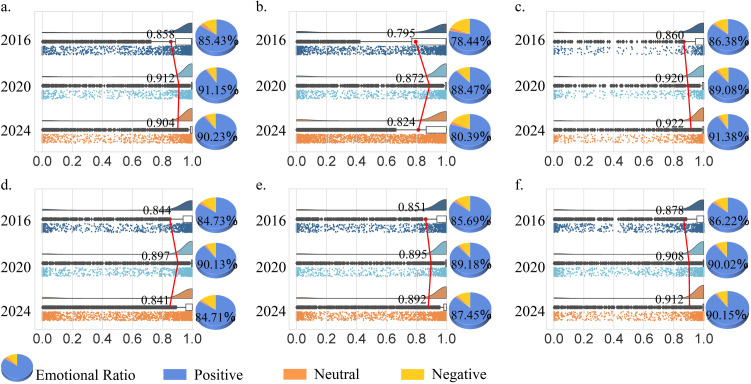
Spatial experience satisfaction for different museum types. a-f represent intangible cultural heritage, history, ethnography, site, art, and natural science museums, respectively.

Overall, the spatial experience satisfaction associated with different museum types showed an upwards trend from 2016 to 2024. Among them, satisfaction with natural science museums continuously increasing throughout the period, whereas the other four types displayed a rising-then-decreasing trend (Fig 7).

Specifically, more than 75% of the visitors expressed positive satisfaction with the spatial experience across all six museum types throughout the study period, although 10–15% of the visitors gave negative evaluations. Moreover, there were significant differences in overall experience satisfaction among museum types. Ethnography museums exhibited the highest satisfaction levels in both 2020 (*Pro* = 0.920) and 2024 (*Pro* = 0.922), whereas history museums consistently received the lowest satisfaction ratings, particularly in 2016 (*Pro* = 0.795).

#### 3.4.2 Detailed satisfaction analysis of the spatial experience of 30 museums.

**Fig 8:**
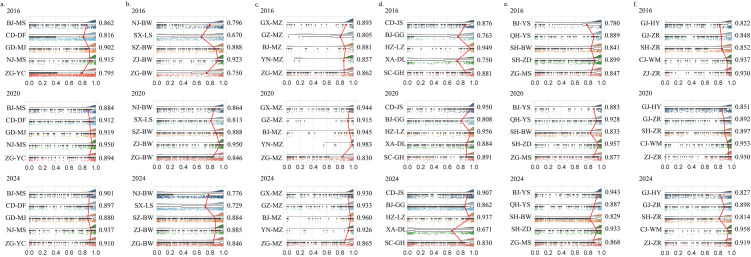
Spatial experience satisfaction of 30 museums. a-f represent intangible cultural heritage, history, ethnography, site, art, and natural science museums, respectively.

The spatial experience satisfaction levels of 30 museums changed significantly from 2016 to 2024 (Fig 8). Of these, 63.33% of the museums exhibited a positive growth trend in satisfaction, while 33.33% showed a rising-then-declining trend. Only the Shanghai Museum (SH-BW) exhibited a consistent downward trend, with its satisfaction score (*Pro* value) decreasing from 0.841 in 2016 to 0.829 in 2024.

Specifically, 1) Among museums with positive growth, approximately 26.67%, such as the Beijing Folk Museum (BJ-MS), experienced continuous growth in spatial experience satisfaction. 2) The Shaanxi History Museum (SX-LS) had the lowest satisfaction in all periods, whereas the Liangzhu Museum (*Pro* = 0.949), Yunnan Ethnic Museum (*Pro* = 0.983), and Beijing Ethnic Culture Palace (*Pro* = 0.960) achieved the highest spatial experience satisfaction in 2016, 2020, and 2024, respectively. 3) In comparison, spatial experience satisfaction was generally greater across museums in 2020, most notably in the Nanjing Folk Museum (NJ-MS) and Qin Shi Huang Mausoleum Museum (XA-DL).

### 3.5 Impact of museum spatial perception themes on public experience satisfaction

**Fig 9:**
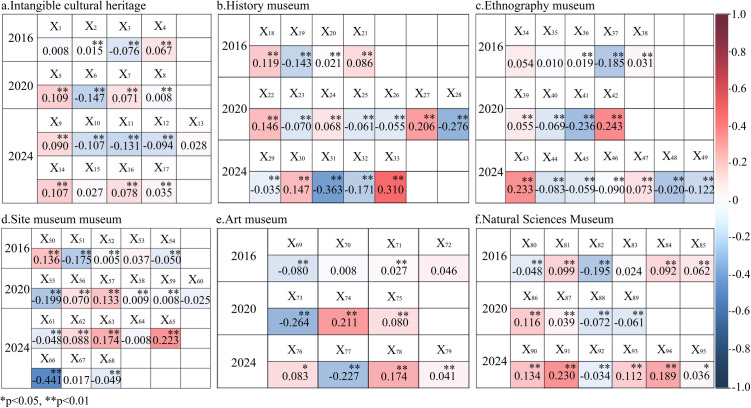
Relationships between spatial satisfaction levels and spatial image themes of six museum types from 2016–2024.

The influence of the perception themes on spatial experience satisfaction for the six types of museums is both dynamic and complex (Fig 9), with 85.26% of the themes showing highly significant correlations with satisfaction levels (*p* < 0.01). Generally, themes positively associated with satisfaction are focused on rich collections, distinct features, immersive experiences, and multidimensional visiting formats, whereas negative experiences are attributed mainly to ticket reservations, value for money, poor environments and services, and frequent changes in exhibition themes.

Specifically, 1) the factors affecting spatial experience satisfaction for the same type of museum are dynamic. For example, in art museums, from 2020–2024, the factors influencing positive visitor experiences shifted from online tour services (*r* = 0.211, *p* < 0.01) to immersive scene construction (*r* = 0.174, *p* < 0.01) and related products (*r *= 0.083, *p* < 0.01). 2) The same perception theme can have different effects on the same type of museum over time. For example, the theme of “reservation and visiting time” in art museums had a significant positive correlation with satisfaction in 2016 (*r* = 0.027, *p* < 0.01), but by 2024, this relationship had shifted to a significant negative correlation (*r* = −0.227, *p* < 0.01). In terms of intensity, the influence of the theme “authenticity of site exhibits” on visitor satisfaction in site museums increased from 0.136 in 2016 to 0.223 in 2024 (*p* < 0.01). 3) The impact of the same theme varies significantly across different museum types over time. For example, in natural science museums, satisfaction was significantly negatively correlated with the theme “simplistic artwork interpretation” (*r* = −0.048, *p* < 0.01), whereas in ethnographic museums, the same theme had a significantly positive correlation with satisfaction (*r* = 0.233, *p* < 0.01).

In summary, the influence of spatial perception themes on spatial experience satisfaction varies across time and museum type, offering valuable insights for formulating dynamic strategies to enhance museum spatial imagery.

## 4 Discussion

This study combined spatial analysis methods, LDA topic modelling, and sentiment analysis to explore online review data from 30 museums (across six types) in China in detail. It revealed the spatio-temporal distribution of visitor preferences, perception themes, satisfaction levels, and their dynamic influencing factors. This interdisciplinary approach opens a new direction for museum space research, overcoming the limitations of traditional static analyses. It offers a visitor behavior research framework from a spatio-temporal evolution perspective, providing new references for optimizing museum space quality.

### 4.1 The influence mechanism of public preference distribution and spatial satisfaction levels

First, the richness of locational resources and regional cultural characteristics are key factors influencing the distribution of visitor density. For example, museums in Beijing, Jiangsu, and Zhejiang, particularly intangible cultural heritage and history museums, continue to attract large numbers of visitors (Fig 3). This is likely closely related to the richness of cultural resources, economic development, and government support for cultural heritage protection policies in these areas [46,47]. For example, Jiangsu Province has promoted its cultural strength strategy, providing policy support for high visitor density in intangible cultural heritage museums [42]. In contrast, ethnography museums in minority regions such as Guizhou and Guangxi also present high visitor density, reflecting the attraction of unique cultural resources and rich ethnic customs in these regions [48,49].

Second, the digital transformation of museums profoundly reshapes public spatial preferences and perceptions of museum spaces. Our findings indicate that public perception themes have gradually shifted from focusing on “objects” (exhibits) to “subjects” (visitor experiences). This shift is closely aligned with the increasing emphasis museums have placed on digital exhibitions, immersive experiences, and cultural interactions in recent years [50,51]. For example, TF-IDF and LDA analyses revealed significant changes in visitor perception themes, particularly in 2024, when the number of perception themes for intangible cultural heritage museums expanded to nine (Fig 6). This reflects an increased demand among visitors for more diverse spatial imagery [52]. This transition from traditional displays to interactive experiences suggests that museums need to continually innovate their spatial design and exhibition formats to better meet the diverse needs of visitors [53].

Moreover, the cultural industry has been significantly impacted by the major public health event, like the COVID-19 pandemic [54]. For example, In 2020, as a key turning point due to the COVID-19 lockdown policies, notable changes occurred in both museum experience satisfaction and visitor distribution patterns. Among the six museum types, four experienced a decline in satisfaction levels after 2020 (Fig 7). However, compared with 2016, the overall satisfaction levels in 2020 were higher, which could be attributed to the psychological compensation mechanism following the pandemic [55]. The impact of COVID-19 also manifested in the weakening of regional museum connections. In 2020, the regional connectivity of Site, Art, and Natural Science museums decreased significantly (Fig 3), a trend closely tied to changes in public health measures and travel patterns [56]. This finding is consistent with UNESCO’s report, which highlighted the severe impact of COVID-19 on museums globally [57]. Furthermore, in contrast to traditional studies that focus on individual museums [58] or regional museums [13], our research revealed that ethnography and natural science museums were relatively less affected during the COVID-19 pandemic, with their satisfaction levels continuing to rise. This underscores the importance of adopting a typological approach when conducting museum research. In conclusion, the impact of COVID-19 on museums has been significant, both negative and positive. However, this profound impact has accelerated the digital transformation of museums [50], fostering deeper connections between digital technologies and cultural artefacts, particularly in intangible cultural heritage museums.

### 4.2 Multi-dimensional public space research framework based on perception and experience theory

**Fig 10:**
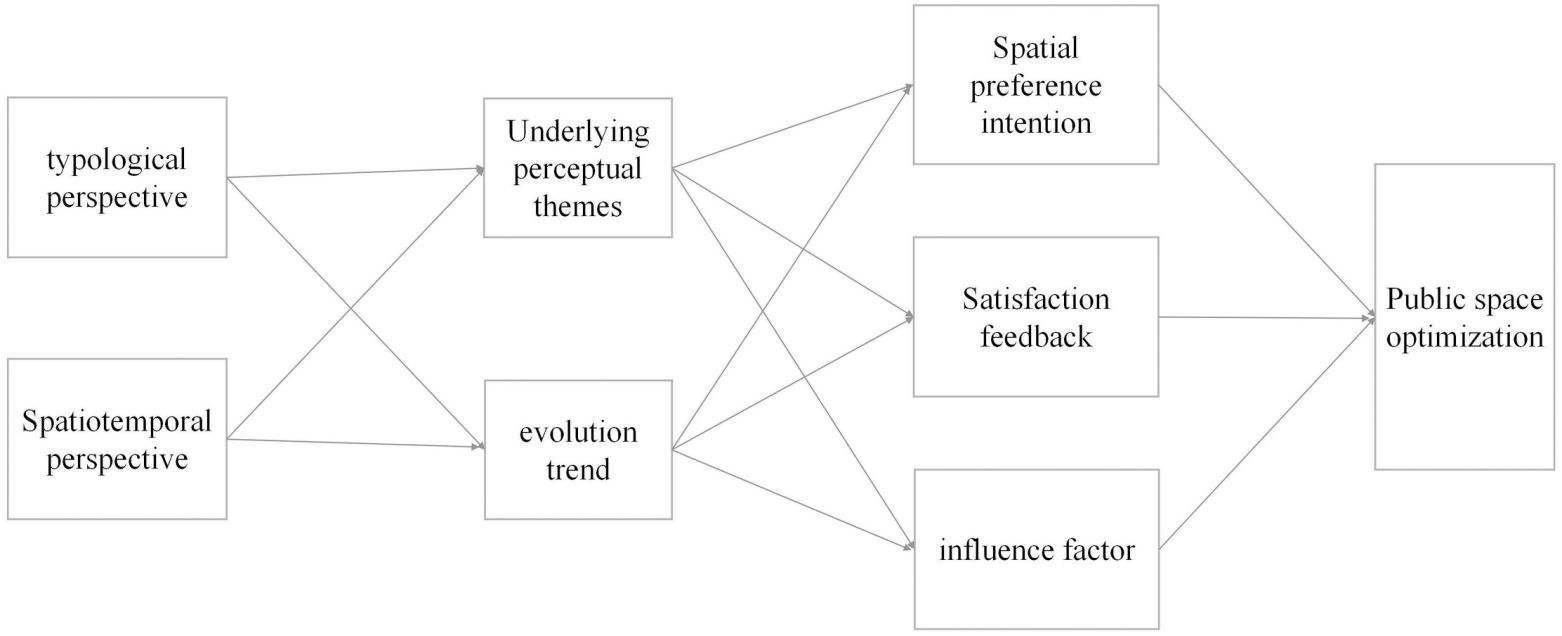
Multidimensional public space theoretical framework.

First, this study introduces a novel theoretical framework for analyzing museum spatial preferences and perceptions by integrating typological and spatio-temporal perspectives (Fig 10). Unlike previous studies that largely rely on static analyses at specific time points or focus on individual museums [5,6,10], our framework captures the dynamic evolution of spatial preferences and perception processes over a long time span (2016–2024). This dynamic approach not only enhances our understanding of visitor behavior but also provides a conceptual shift toward understanding public space usage in a more holistic and temporally sensitive manner.

Second, by combining GIS spatial analysis with machine learning techniques—specifically the TF–IDF and LDA models—our framework unveils the latent visitor perception themes embedded within large-scale textual data and reveals their evolving trends [59]. This integration represents a significant theoretical contribution, as it offers new insights into how public perceptions are formed and transformed over time. In contrast to traditional surveys or interviews [19,20], our approach leverages naturally occurring social media reviews, which yield relatively more authentic public feedback, reduce biases such as self-selection [60], and facilitate the analysis of extensive datasets.

Moreover, this study fills the research gap concerning the relationship between museum perceptions and satisfaction. While many studies have focused on museum imagery or satisfaction separately [13,61], this study, which uses Pearson correlation analysis, reveals significant typological differences and spatio-temporal evolution in how spatial imagery affects satisfaction. For example, in art museums, the theme “reservation and visiting time” was positively correlated with satisfaction in 2016 (*r* = 0.027, *p* < 0.01), but this correlation became negative by 2024 (*r* = −0.227, *p* < 0.01). This suggests that museum service experiences need to be adjusted according to changing visitor demands [2].

In summary, the framework based on perception and experience provides method reference for future research on the dynamic interaction between public space and public experience, and suggests potential ways to optimize museum spaces and other public cultural places.

### 4.3 Multidimensional strategies for optimizing different types of museums

The practical significance of this study lies in providing museum managers with empirical evidence to optimize spatial design and service strategies. First, the results indicate that the reservation system and frequent changes in exhibition themes are the primary factors influencing negative visitor experiences. Particularly in site museums, poor crowd management and ticketing systems led to heightened negative emotions (*r* = −0.441, *p* < 0.01), indicating the need for more refined visitor flow management measures, such as time-slot ticketing and online reservations [2].

Second, the study demonstrated that cultural experiences and immersive scene construction are crucial for enhancing visitor satisfaction. In history museums, satisfaction level was significantly positively influenced by digital exhibitions and cultural dissemination experiences (*r* = 0.310, *p* < 0.01). For example, the Beijing Art Museum successfully enhanced visitor engagement with exhibitions by integrating virtual reality technology and receiving highly positive feedback [62]. This finding suggests that museums can introduce more modern technologies to increase the interactivity and appeal of exhibitions, thereby improving public cultural experiences.

Additionally, the study highlights the importance of surrounding service facilities in enhancing visitor satisfaction. Particularly in natural science museums, the adequacy of surrounding facilities was closely related to visitor satisfaction (*r* = 0.230, *p* < 0.01). This finding suggests that museum managers should prioritize improving surrounding services, such as transportation, dining, and leisure facilities, to enhance the overall cultural tourism experience [6].

### 4.4 Limitations

Despite these findings, this study has several limitations. 1) The data sources for this study should be expanded. It relies primarily on online review data from platforms such as Dianping and Ctrip, which may not fully capture the experiences of certain visitor groups, particularly elderly and international tourists. Future research should consider incorporating other social media platforms, such as Weibo and Zhihu, to gather more diverse feedback and enhance the representativeness of the study. 2) Although this study includes 30 museums across six categories, the sample size is relatively small, which limits the generalizability of the findings, particularly when analysing cross-regional and cross-type differences among museums. Future research should aim to increase the sample size and integrate visitor behavior tracking data to further analyse the interaction between perceptions and actual behavior. 3) With the continuous advancement of museum digitalization, future studies should explore more innovative models of digital exhibitions and visitor interaction. How to leverage digital methods to enhance museum spatial experiences and address operational challenges remains a valuable direction for future exploration. 4) Data from September to December 2024 were supplemented with 2023 data to analyse the latest trends. As the primary focus of this study is evolutionary trends, this data supplementation has a limited impact on the overall trend analysis. Future research should aim to collect complete annual data to further improve the accuracy of conclusions.

## 5 Conclusions

By integrating GIS spatial analysis and machine learning techniques such as LDA topic modelling, this study delved deeply into the dynamic evolution of museum spatial perception themes. This interdisciplinary approach not only overcomes the limitations of traditional static analysis but also reveals the key driving factors of visitor satisfaction across different types of museums between 2016 and 2024. By combining a spatio-temporal perspective with typology, this research provides a new approach for understanding the complexity of visitor needs and reveals the profound influence of museum spatial imagery on visitor satisfaction, offering evidence for improving museum spatial quality. Our main contributions are as follows:

1) Museum public spatial preferences have evolved from “single-core” to “multi-core” patterns, with eastern coastal regions and minority-dominated areas in central and western China becoming visitor concentration zones for different types of museums. This finding indicates that regional cultural resources and policies significantly impact visitor behavior.
2) From 2016 to 2024, spatial imagery perceptions of museum gradually shifted from focusing on “objects” to “people,” with notable differences in imagery themes across 6 museum types. Intangible cultural heritage museums experienced the broadest expansion of spatial imagery perception, especially in 2024 (*K* = 9), whereas art museums experienced the lowest expansion throughout the period (*K* = 4). Additionally, visitors to intangible cultural heritage, history, and site museums were more focused on cultural education themes, whereas visitors to art, ethnography, and natural science museums were more inclined toward local cultural identity and entertainment attributes.
3) The experience satisfaction levels of six types of museums from 2016–2024 were evaluated. Overall, the satisfaction levels of these museums showed an increasing trend, with the highest level of satisfaction among ethnographer museums in 2024 (*Pro* = 0.922) and the lowest level of satisfaction among history museums (average *Pro* = 0.83). At the individual museum level, approximately 63.33% of museums showed positive growth in spatial experience satisfaction, with only the Shanghai Museum exhibiting a continuous decline.
4) There is a dynamic and complex relationship between museum spatial perception themes and visitor satisfaction. Approximately 85.26% of museum spatial imagery is highly correlated with satisfaction (*p* < 0.01). The impact of different perceptions on satisfaction varies significantly across periods and museum types. For example, in art museums, the factors influencing positive experiences shifted from online tour services in 2020 (*p* < 0.01) to immersive scene construction in 2024 (*p* < 0.01). Conversely, “reservations”, “poor service” and “frequent changes in exhibition themes” were the main factors reducing satisfaction levels. These findings provide a basis for museums to dynamically adjust their spatial imagery.

In summary, this study not only provides data support for museum spatial optimization and management but also offers new insights into the sustainable development of cultural heritage through innovative methods. In the future, museums should continue to enhance their spatial imagery and visitor experiences by integrating technological innovation with cultural diversity to promote the preservation and dissemination of cultural heritage.

## Supporting information

S1 File All online review text data. (XLSX)
